# Combining Metabolomics and Interpretable Machine Learning to Reveal Plasma Metabolic Profiling and Biological Correlates of Alcohol-Dependent Inpatients: What About Tryptophan Metabolism Regulation?

**DOI:** 10.3389/fmolb.2021.760669

**Published:** 2021-11-08

**Authors:** Xiuqing Zhu, Jiaxin Huang, Shanqing Huang, Yuguan Wen, Xiaochang Lan, Xipei Wang, Chuanli Lu, Zhanzhang Wang, Ni Fan, Dewei Shang

**Affiliations:** ^1^ Department of Pharmacy, The Affiliated Brain Hospital of Guangzhou Medical University (Guangzhou Huiai Hospital), Guangzhou, China; ^2^ Guangdong Engineering Technology Research Center for Translational Medicine of Mental Disorders, Guangzhou, China; ^3^ Department of Substance Dependence, The Affiliated Brain Hospital of Guangzhou Medical University (Guangzhou Huiai Hospital), Guangzhou, China; ^4^ Department of Medical Sciences, Guangdong Provincial People’s Hospital, Guangdong Academy of Medical Sciences, Guangzhou, China; ^5^ Guangzhou Rely Medical Diagnostic Technology Co. Ltd., Guangzhou, China

**Keywords:** alcohol dependence, metabolic profiling, biological correlate, metabolomics, machine learning, tryptophan metabolism, orthogonal partial least squares-discriminant analysis, metabolic pathway

## Abstract

Alcohol dependence (AD) is a condition of alcohol use disorder in which the drinkers frequently develop emotional symptoms associated with a continuous alcohol intake. AD characterized by metabolic disturbances can be quantitatively analyzed by metabolomics to identify the alterations in metabolic pathways. This study aimed to: i) compare the plasma metabolic profiling between healthy and AD-diagnosed individuals to reveal the altered metabolic profiles in AD, and ii) identify potential biological correlates of alcohol-dependent inpatients based on metabolomics and interpretable machine learning. Plasma samples were obtained from healthy (*n* = 42) and AD-diagnosed individuals (*n* = 43). The plasma metabolic differences between them were investigated using liquid chromatography-tandem mass spectrometry (AB SCIEX^®^ QTRAP 4500 system) in different electrospray ionization modes with scheduled multiple reaction monitoring scans. In total, 59 and 52 compounds were semi-quantitatively measured in positive and negative ionization modes, respectively. In addition, 39 metabolites were identified as important variables to contribute to the classifications using an orthogonal partial least squares-discriminant analysis (OPLS-DA) (VIP > 1) and also significantly different between healthy and AD-diagnosed individuals using univariate analysis (*p*-value < 0.05 and false discovery rate < 0.05). Among the identified metabolites, indole-3-carboxylic acid, quinolinic acid, hydroxy-tryptophan, and serotonin were involved in the tryptophan metabolism along the indole, kynurenine, and serotonin pathways. Metabolic pathway analysis revealed significant changes or imbalances in alanine, aspartate, glutamate metabolism, which was possibly the main altered pathway related to AD. Tryptophan metabolism interactively influenced other metabolic pathways, such as nicotinate and nicotinamide metabolism. Furthermore, among the OPLS-DA-identified metabolites, normetanephrine and ascorbic acid were demonstrated as suitable biological correlates of AD inpatients from our model using an interpretable, supervised decision tree classifier algorithm. These findings indicate that the discriminatory metabolic profiles between healthy and AD-diagnosed individuals may benefit researchers in illustrating the underlying molecular mechanisms of AD. This study also highlights the approach of combining metabolomics and interpretable machine learning as a valuable tool to uncover potential biological correlates. Future studies should focus on the global analysis of the possible roles of these differential metabolites and disordered metabolic pathways in the pathophysiology of AD.

## Introduction

Alcohol use disorder (AUD), as described in the fifth edition of the Diagnostic and Statistical Manual of Mental Disorders (DSM–5), is a chronic, relapsing brain disorder including alcohol abuse and alcohol dependence (AD) (Takahashi et al., 2017). AUD presents a potential public health crisis worldwide. According to the global status report on alcohol and health 2018 (World Health Organization, 2018), about three million deaths worldwide and 132.6 million disability-adjusted life years (DALYs) were attributable to the harmful use of alcohol in 2016. AD, defined in the International Classification of Diseases (ICD–11), is “*a disorder of regulation of alcohol use arising from repeated or continuous use of alcohol*” (Saunders et al., 2019). Additionally, there is good concordance in the diagnosis of AD between ICD-10, ICD-11, and DSM-IV (Lago et al., 2016). AD—also known as alcoholism or alcohol addiction—is characterized by compulsive alcohol seeking and taking behaviors, a loss of self-control in limiting intake, and the emergence of an alcohol withdrawal syndrome (including anxiety, agitation, delirium, nightmares, and insomnia) in the absence of the drug (Hall and Zador, 1997; Koob and Le Moal, 1997; Roberto et al., 2021). AD can also induce psychiatric comorbidity, including depressive and anxiety disorders, and, conversely, the comorbid psychiatric disorders can aggravate the severity of alcohol use patterns (Fein, 2015).

The pathophysiological mechanisms of AD have not been fully elucidated. Considerable evidence has suggested the disruption in the mesolimbic dopamine system (an essential part of the reward systems) or the alcohol-associated changes in the hypothalamic-pituitary-adrenal (HPA)-axis in AD (Dai et al., 2007; Hillemacher, 2011; Engel and Jerlhag, 2014). Other central nervous systems (e.g., endogenous opioid, the GABAergic, glutamatergic, and serotonergic) have also been described (Hillemacher, 2011). Novel evidence, such as genetic and epigenetic alterations and the gut-to-brain interactions in AD, has recently emerged (D'Addario et al., 2017; Leclercq et al., 2014; Meng et al., 2019). Alcohol could affect many neurotransmitters and modulators within the brain. For example, tryptophan, an extensively studied amino acid related to alcohol and alcoholism, plays an important role in regulating neuropsychiatric disorders and commonly serves as a precursor for the biosynthesis of multiple biologically or neurologically active substances. Fortunately, the metabolomics approach gives us a chance to study the metabolic alterations of AD. Therefore, this approach provides new insights into the physiological alterations in AD.

Metabolomics is a high-throughput tool for quantitatively analyzing the small-molecule metabolites in biospecimens such as blood, tissue, urine, or saliva (Cheng et al., 2018). It has been increasingly applied to discovering potential biomarkers and related metabolic pathways (Johnson et al., 2016), the investigations of polypharmacological mechanisms of drug combination therapy (Li et al., 2021a), and the host response to the drug therapy (Wang et al., 2018; Li et al., 2020), and explorations of complicated pathophysiologic mechanisms of diseases (Johnson et al., 2016; Wu et al., 2020). Generally, the widely targeted metabolomics method can achieve accurate quantification of targeted metabolites by defining ion-pairs information derived from untargeted metabolomics or obtained from relevant references and existing mass spectrum public databases (Heikkinen et al., 2019; Zhou et al., 2021). Recently, several human metabolomics studies have been reported to investigate the metabolic profiles associated with unhealthy alcohol consumption (such as AUD and AD) based on untargeted/targeted mass spectrometry (MS) and proton nuclear magnetic resonance (^1^H-NMR) spectroscopy approaches (Obianyo et al., 2015; Mostafa et al., 2016; Hinton et al., 2017; Mostafa et al., 2017; Irwin et al., 2018). Particularly, Mittal and Dabur (Mittal and Dabur, 2015) reported the urine metabolic signature of chronic AD before and after treatment with Tinospora cordifolia aqueous extract through the targeted and untargeted liquid chromatography-tandem mass spectrometry (LC-MS/MS) method. However, few studies about the alcohol-associated metabolism changes in the blood plasma in AD patients referring to the use of MS-based metabolomics tools have been reported. Our study, therefore, fills this gap.

Machine learning, as a field of artificial intelligence (AI), has achieved rapid progress in recent years and is gradually emerging in the field of metabolomics due to a diverse spectrum of algorithms, such as the artificial neural network (ANN), random forest (RF), support vector machine (SVM), and genetic algorithms (Liebal et al., 2020). However, as an early developed machine learning method, ANN and other subsequently developed deep learning algorithms are quite uninterpretable and criticized as “black boxes” (Krittanawong et al., 2019), which limited the applicability of many AI-based approaches to medicine. The interpretable “glass-box” machine learning approaches (e.g., linear regression, logistic regression, and decision trees) make AI trustworthy through human-friendly explanations (Rai, 2020). For example, the tree-based decision tree algorithm is interpretable by splitting each feature based on certain cut-off values, thus telling us how the decision is taken starting from the tree’s root node to its leaf nodes at the bottom. Notably, the RF algorithm, an ensemble learning method using the bagging technique, combines multiple decision tree models, thus reducing the variance and greatly boosting the performance (Yaman and Subasi, 2019). However, random forests are typically treated as “black-box” models losing a degree of interpretability as their decisions may be opaque (Borstelmann, 2020). Decision tree-based machine learning has been an emerging approach in metabolomics for disease discrimination and biomarker detection (Allalou et al., 2016; Shao et al., 2017; Murata et al., 2019). In addition, comparing with linear regression and logistic regression models, decision trees are more successful in processing nonlinear relationships between input features and outcomes, particularly suitable for these situations existing in metabolomics due to the nonlinear and dynamic disease states (Zhu et al., 2021c).

This study aimed to reveal the plasma metabolic profiles of AD patients and identified the significantly distinctive metabolites for AD discrimination using a widely targeted metabolomics method based on LC-MS/MS. We also investigated the significantly enriched metabolic pathways involved in AD, together with the distinctive metabolites detected in those pathways. Further, as an interpretable supervised machine learning algorithm, a decision tree classifier was built for AD discrimination and identifying the most important distinctive metabolites, being regarded as potential biological correlates. Notably, we mainly focused on the tryptophan metabolism regulation or abnormality in AD. All the findings of our study in this field may benefit researchers by illustrating the underlying molecular mechanisms of AD.

## Materials and Methods

### Subjects

A total of 85 individuals, between 18 and 65 years of age, were recruited. The participants comprised 43 AD patients (AD group) and 42 healthy controls (HC group). AD patients were recruited from the Affiliated Brain Hospital of Guangzhou Medical University and healthy controls were enrolled through advertisements. The patients were enrolled in the AD group if they were clinically diagnosed as AD according to the DSM–IV diagnostic criteria and had the Clinical Institute Withdrawal Assessment for Alcohol, Revised (CIWA-Ar) scores less than ten. The exclusion criteria used for the AD group included: 1) other mental disorders which met DSM-IV-TR criteria (excluding nicotine dependence and AD); 2) a history of psychoactive substances (excluding alcohol and nicotine) use; 3) serious comorbid somatic diseases (e.g., heart failure and severe liver and kidney diseases); 4) a history of neurological disorders (e.g., epilepsy, neurosurgery, and severe head trauma with or without loss of consciousness); 5) pregnancy. Healthy controls had no current or history of mental disorders, no familial history of mental disorders, and no severe physical disease. Exclusion criteria for healthy controls were: 1) any known brain organic diseases; 2) a history of head trauma with loss of consciousness; 3) any unstable physical disease. All subjects recruited had not drunk alcohol since they were admitted to the hospital, and were screened for substance use other than alcohol and tobacco through urine drug testing. The study was conducted in compliance with the guidelines of the Helsinki Declaration and was approved by the independent Ethics Committee of the Affiliated Brain Hospital of Guangzhou Medical University (ethics number: 2019003); all participants provided informed consent.

### Chemicals, Reagents, and Equipment

Methanol, acetonitrile, ammonium acetate (NH_4_Ac), and aqueous ammonia (NH_4_OH) were all high-performance liquid chromatography (HPLC)-grade and were purchased from Thermo Fisher Scientific (Waltham, MA, United States). All the experiments were conducted on an ultra-high performance liquid chromatography (UHPLC) system including two Shimadzu LC-30AD pumps, a SIL-30AC auto-sampler, and a CTO-20AC column oven (Shimadzu Corporation, Kyoto, Japan), and coupled with QTRAP 4500 mass spectrometer (AB SCIEX, CA, United States). The PLRP-S column (3.0 µm, 150 mm × 2.1 mm) was purchased from Agilent Technologies (Santa Clara, CA, United States).

### Plasma Sample Collection and Sample Preparation

Metabolomic analysis was conducted in plasma samples, which were collected from all the participants. The plasma was separated from the peripheral blood samples in EDTA tubes by centrifuging at 3,000 rpm for 10 min at 4°C and was immediately stored at −80°C until future metabolomics analysis to minimize the metabolic degradation process. The plasma samples (150 μl) were treated with a certain amount of ice-cold methanol (stored at −80°C for approximately 5 h). After vortexing for 2 min, the pooled samples were stored at −80°C for 1 h and centrifuged future at 14,000 × *g* for 10 min at 4°C. The supernatant was transferred and then concentrated to dryness under a vacuum. Before the metabolomics analysis, a 150 μl mixed solution of acetonitrile/H_2_O (1:1, *v*/*v*) taken as the reconstitution solution was added to the dry extract samples. The pooled quality control (QC) sample was prepared by mixing an equal aliquot (40 μl) of each plasma sample to verify the methodology of the metabolomics analysis. One QC sample was inserted at every ten samples in an analysis batch consisting of 11 QC samples in total.

### LC-MS/MS-Based Metabolomics Method

Chromatographic separation was performed on an Agilent PLRP-S column using a flow rate of 0.35 ml/min. The temperatures of the autosampler and column were kept at 4 and 40°C, respectively. The mobile phase A consisted of H_2_O/acetonitrile (95:5, *v*/*v*) with 20 mmol/l NH_4_AC and 20 mmol/l NH_4_OH (pH = 9.0), and the mobile phase B was acetonitrile. The total elution time was 15 min for the gradient program, of which the details were as follows: 2% B was held at the initial 0.2 min, then linearly increased to 90% B from 0.2 to 9 min, next held 90% B for 2 min, and finally returned to 2% B in 0.1 min, following by equilibration at 2% B for 3.9 min.

In this study, we acquired data of metabolites based on the defined multiple reaction monitoring (MRM) ion-pairs of interest collected from related references published elsewhere (Moriarty et al., 2011; Zhu et al., 2011; Yuan et al., 2012; Fuertig et al., 2016; Tudela et al., 2016; Takada et al., 2018; Wang et al., 2019), the Human Metabolome Database (HMDB, https://www.hmdb.ca), and AB SCIEX™ (refer to: https://sciex.com/content/dam/SCIEX/pdf/tech-notes/life-science-research/metabolomics/Targeted-Mx-method_RUO-MKT-02-13259-A.pdf). The electrospray ionization (ESI) source was operated in the positive ion (ESI+) and negative ion (ESI−) modes, respectively, with the following main mass spectrometric parameters: capillary temperature, 475°C (ESI+ and ESI− modes); ion spray voltage, 5500 V (ESI+ mode) and −4,500 V (ESI− mode); collision gas, “medium” (ESI+ and ESI− modes); curtain gas, 25 psi (ESI+ and ESI− modes); ion source gas1 and gas2, 45 psi (ESI+ and ESI− modes). The reconstituted supernatants were injected twice for both ESI+ and ESI− mode analyses, and the injection volume was 5 μl for all samples.

### LC-MS/MS Data Processing and Bioinformatic Analysis

Data processing, such as integrating the peaks’ areas, was performed using the MS quantitation software—MultiQuant™ Software (version 3.0.3, AB SCIEX, CA, United States). The metabolomic data analysis included heatmap clustering and multivariate statistical analysis methods such as principal component analysis (PCA) and orthogonal partial least squares discriminant analysis (OPLS-DA). The data analysis and interpretation, such as metabolic pathway analysis, were conducted based on the MetaboAnalyst (V5.0) platform (https://www.metaboanalyst.ca) (Xia et al., 2009; Pang et al., 2021). The comparison of relative levels of metabolites between the two groups was displayed in a heatmap with hierarchical clustering. The variable influence on projection (VIP) values presents the overall influence of each *x*-variable in the OPLS-DA model on *y*-variables. The two groups’ differential metabolites were identified using a statistically significant threshold value of VIP > 1 (*Q*-value < 0.05) obtained from the OPLS-DA model and univariate analysis (Lee et al., 2020; Li et al., 2021b). The information of the identified distinctive metabolites was then input to the MetaboAnalyst platform to obtain the significantly perturbed metabolic pathways related to AD.

### Discrimination of Alcohol-Dependent Inpatients Using Decision Tree Classifier

The machine learning dataset consisted of the entire samples from AD and HC groups (i.e., labels). The distinctive metabolites obtained from the OPLS-DA model were treated as features for decision tree construction to obtain credible results. Before analysis, the peak areas of those metabolites were rescaled into the range of 0–1 using min-max normalization to minimize the influence of changes in the response of LC-MS/MS. The formula for a min-max normalization is: *x*_new_ = (*x*–*x*_min_)/(*x*_max_–*x*_min_). Subsequently, 80% of the data (i.e., 68 samples) were randomly selected as the “training set” to develop the decision tree classifier model; the remaining 20% (i.e., 17 samples) went into the “test set” for model validation. Based on the training set, the feature importance scores provided by the “feature_importance_” attribute of the decision tree were used for feature selection; thereafter, the optimal parameters of our model were filtered by hyperparameter optimization using the tool of ten-fold cross-validation in GridSearchCV. The evaluation metrics for the developed model included confusion matrix, accuracy, precision, sensitivity (also known as recall), f1 score, the receiver operating characteristic (ROC) curve plot, and the area under the curve (AUC). Finally, an interpretable decision tree diagram and a decision boundary were created to visualize the fitted model.

All the data analyses, model construction, model evaluation, and visualizations were performed using Python (version 3.8.5, https://www.python.org) and related packages, including the scikit-learn package (version 0.23.2, https://scikit-learn.org/stable/index.html), seaborn package (version 0.11.0, https://seaborn.pydata.org), pandas package (version 1.1.3, https://pandas.pydata.org), NumPy package (version 1.19.2, https://numpy.org), matplotlib package (version 3.3.2, https://matplotlib.org), and scipy package (version 1.5.2, https://www.scipy.org). This was based on the Jupyter Notebook (version 6.1.4, https://jupyter.org), launching from the Anaconda Navigator (version 4.9.2, https://www.anaconda.com, Anaconda Inc., Austin, TX, United States).

## Results

### Clinical Characteristics of Subjects

The basic characteristics of the participants are listed in Table 1. There was no statistically significant difference in age among the two groups (*p* = 0.604). Though only male subjects were enrolled in this study, the two groups were gender- and age-matched. Nevertheless, the smoking and alcohol intake frequencies between the two groups were significantly different (*χ* ^2^ = 9.027, *p* = 0.011 and *χ* ^2^ = 60.262, *p* < 0.001, respectively). As for the AD group, the interval between last alcohol intake and blood draw was (7.51 ± 5.68) days, and 25 AD patients had low alcohol withdrawal symptoms with the CIWA-Ar scores of one to seven. The most prescribed drugs among the patients before their blood sampling were ranked as follows: fat- and water-soluble vitamins (86.05%), KCl (76.74%), oxazepam (62.79%), vitamin B_1_ (55.81%), diazepam (53.49%), trivitamins B (44.19%), and omeprazole (37.21%), according to the number of patients taking medications.

**TABLE 1:** The demographic and clinical information of subjects.

Items	AD group (*n* = 43)	HC group (*n* = 42)
Gender	43 male subjects; no female subject	42 male subjects; no female subject
Age (years)^a^		
Median	44	47
Minimum–Maximum	24–58	31–65
CIWA-Ar score		
Median	1	NA
Minimum–Maximum	0–7	NA
Alcohol intake (*n*, %)^b^		
Almost everyday	36 (83.72)	2 (4.76)
One to three times per week	4 (9.30)	6 (14.29)
Two to four times per month	1 (2.33)	0 (0)
One time or less per month	2 (4.65)	34 (80.95)
Smoking (*n*, %)^b^		
Smokers	41 (95.35)	30 (71.43)
Non-smokers	2 (4.65)	10 (23.81)
Ex-smokers	0 (0)	2 (4.76)

a Mann-Whitney *U* test, *p* > 0.05.

b Chi-square test, *p* < 0.05.

AD, alcohol dependence patients; HC, healthy controls; CIWA-Ar, the Clinical Institute Withdrawal Assessment for Alcohol, Revised; NA, not available.

### Method Validation Using QC Samples

**FIGURE 1:**
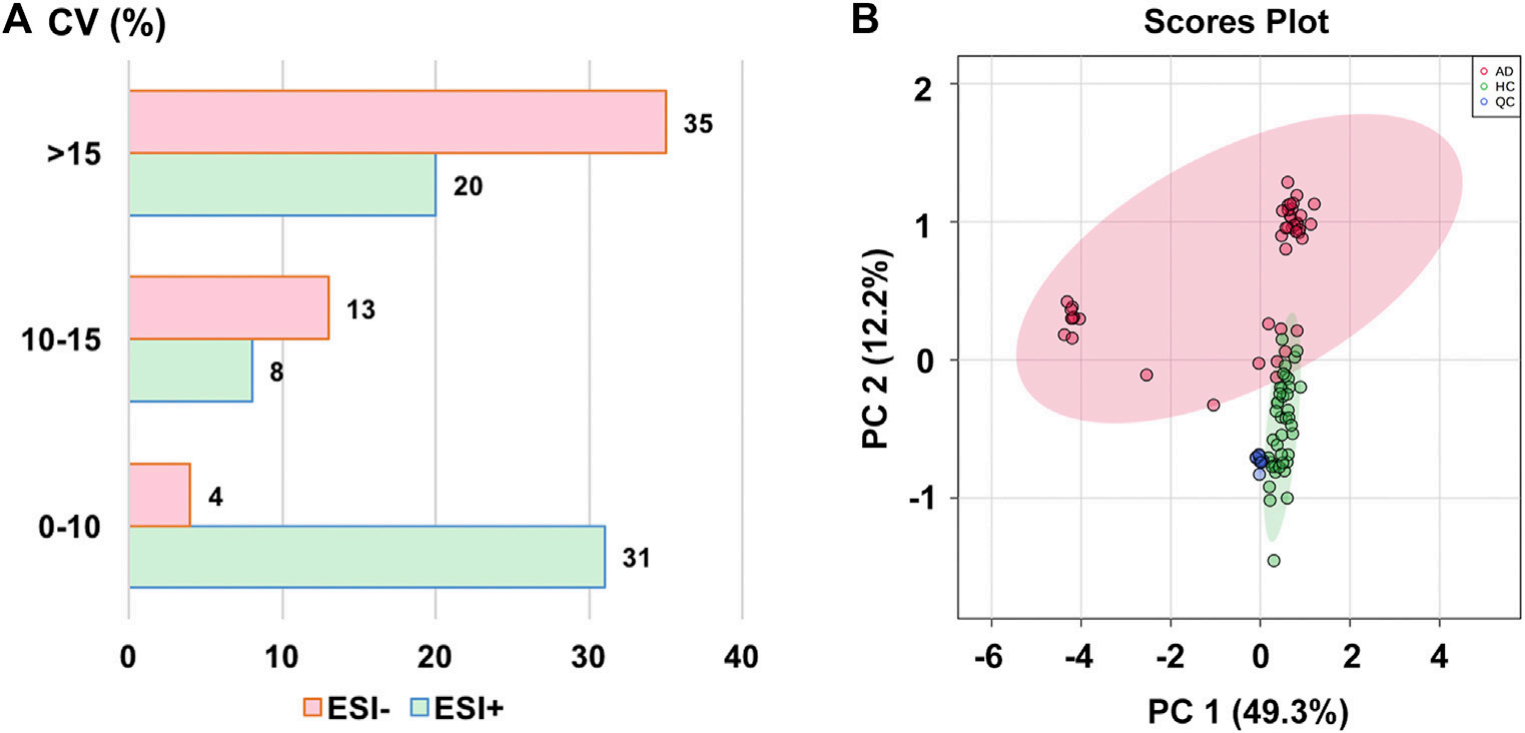
**(A)** The distribution of coefficient of variance (CV) in different electrospray ionization modes; **(B)** The principal component analysis (PCA) score plot with semi-transparent confidence intervals of the healthy controls (HC group) and alcohol dependence patients (AD group). Note. QC, quality control.

The stability of the analytical method has been investigated by using the pooled QC samples before analysis. To develop the metabolomics method, 163 ion-pairs corresponding to 160 compounds of interest were selected. Out of these, 59 ion-pairs (i.e., 59 compounds) were included in the ESI+ mode and 52 ion-pairs (i.e., 52 compounds) in the ESI− mode for semi-quantitative detection, respectively, after manually retaining the only metabolite ion-pair with the best peak performance and removing the compounds with poor peak shape or low response in the peak area (Supplementary Table S1). As a measure of variability, the coefficients of variance (CV) (also known as relative standard deviations) of all these 111 semi-quantitatively measured metabolites’ peak areas in the QC samples were calculated with values of less than 25% (median value, 14.14%), indicating that the metabolomics method was stable and repeatable, and fulfilled the requirements of subsequent metabolomic detection (Solanki et al., 2020). The distribution of CV values is shown in Figure 1A. Figure 1B presents that all the 11 QC samples were tightly located in the PCA score plot, further verifying the excellent repeatability of our analytical method.

### Identification of Differential Metabolites

**FIGURE 2:**
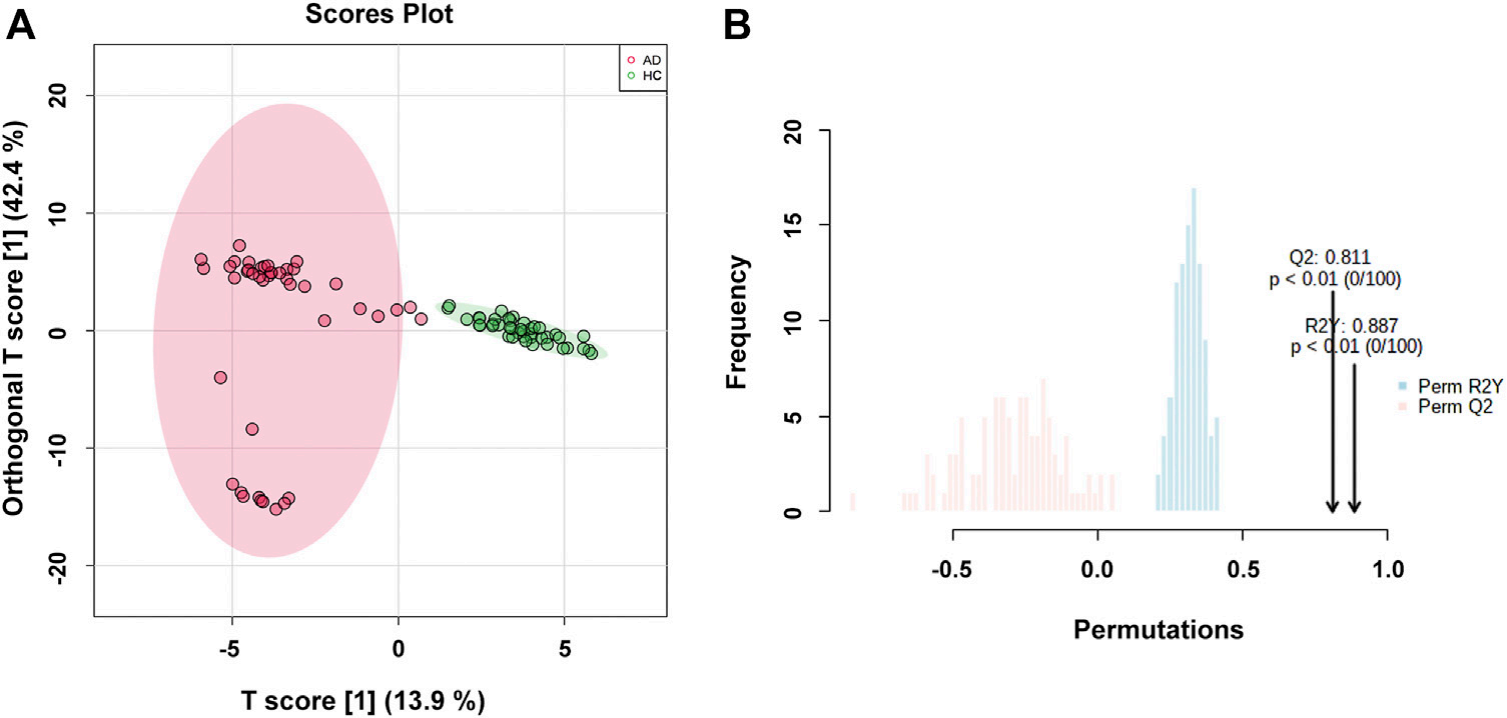
**(A)** The orthogonal partial least squares discriminant analysis (OPLS-DA) score plot with semi-transparent confidence intervals of the healthy controls (HC group) and alcohol dependence patients (AD group); **(B)** The permutation test with permutation number of 100 for the OPLS-DA model.

The OPLS-DA model was used to compare the metabolic profiling differences between the AD group and the HC group. As shown in Figure 2A, the horizontal component of the score plot of the OPLS-DA model displayed obvious discrimination among the HC and AD groups. In contrast, there existed a certain variation within the AD group as captured by the vertical dimension. A 100-iteration permutation test was conducted for validation of the classification performance of the OPLS-DA model with the fit metrics values of R^2^Y = 0.887 (*p* < 0.01) and Q^2^ = 0.811 (*p* < 0.01), indicating that the computed OPLS-DA model was reliable and robust due to avoiding overfitting (Figure 2B) (Westerhuis et al., 2008; Triba et al., 2015; Mo et al., 2021).

**FIGURE 3:**
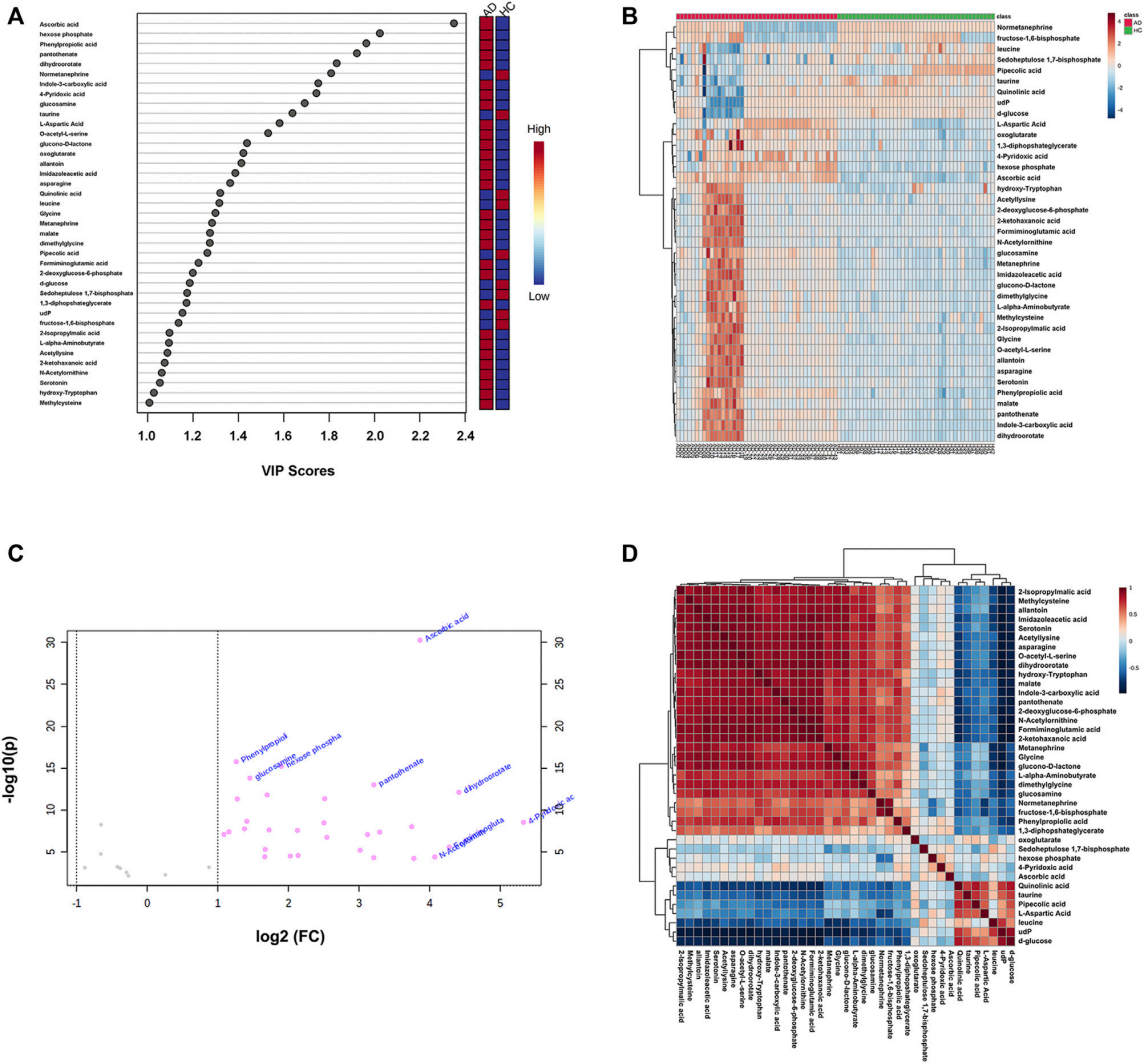
**(A)** The variable influence on projection (VIP) score plot of each distinctive metabolite (VIP > 1 and *Q*-value < 0.05) between the healthy controls (HC group) and alcohol dependence patients (AD group). The color in the block represents the up-regulation (red) or down-regulation (blue) of metabolites. **(B)** A hierarchical clustering heatmap of the 39 identified significantly differential metabolites (represented by rows) among the HC and AD samples (represented by columns). The color in the map displays the relative abundance of metabolites using normalized intensity data. **(C)** The volcano plot is based on fold change (FC) (AD group *vs.* HC group) and −log_10_(*p*) values, highlighting 30 differential metabolites with FC values above two. **(D)** A hierarchical clustering heatmap of the correlations among the differential metabolites in the AD group based on Pearson’s correlation coefficient.

After screening with VIP > 1 and *Q*-value < 0.05 [i.e., *p*-value < 0.05 of Student’s *t*-test after false discovery rate (FDR) adjusting, see Supplementary Table S2], 39 potential differential metabolites were identified, containing 19 metabolites in ESI+ mode and 20 metabolites in ESI− mode. The rank of VIP score of each abovementioned metabolite is presented in Figure 3A. Among the differential metabolites related to AD, indole-3-carboxylic acid, quinolinic acid, hydroxy-tryptophan, and serotonin were of our interest, involving in the tryptophan metabolism along the indole, kynurenine, and serotonin pathways. Nine differential metabolites were significantly downregulated in AD, including normetanephrine, taurine, quinolinic acid, leucine, pipecolic acid, d-glucose, sedoheptulose 1,7-bisphosphate, udP, and fructose-1,6-bisphosphate; whereas the remaining were significantly upregulated. There was a noticeable metabolite difference between the two groups, visualizing in the hierarchical clustering heatmap of these identified significantly differential metabolites (Figure 3B). Notably, volcano plot analysis revealed that a total of 30 differential metabolites obtained fold change (FC) values above two (e.g., 4-pyridoxic acid, dihydroorotate, formiminoglutamic acid, N-acetylornithine, and ascorbic acid), highlighting the levels of which were significantly upregulated in the AD group compared with those in the HC group (Figure 3C). A hierarchical clustering heatmap analyzed by Pearson’s correlation coefficient was also drawn to display the correlations among these differential metabolites in the AD group (Figure 3D).

### Metabolic Pathway Analysis

**FIGURE 4:**
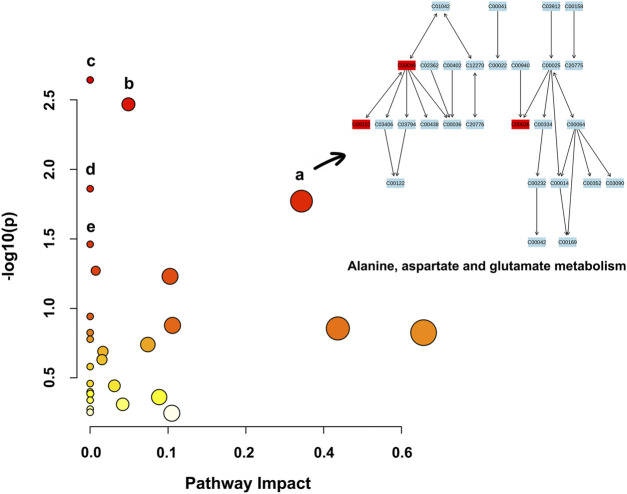
Metabolic pathway enrichment analysis based on the identified distinctive metabolites. Each bubble in the plot represents a metabolic pathway. The size of the bubble indicates the influence of this pathway; the larger the size, the greater the impact values. The color of the bubble indicates the different levels of −log_10_(*p*) values; the darker the color, the more significant the metabolic pathway. The Cxxxxx symbols in the nodes are Kyoto Encyclopedia of Genes and Genomes (KEGG, available at: https://www.kegg.jp/kegg/) database C numbers, serving as identifiers for metabolites in the above pathways (red blocks represent the detected metabolites; blue blocks represent the undetected metabolites). Note. a, alanine, aspartate and glutamate metabolism; b, histidine metabolism; c, arginine biosynthesis; d, aminoacyl-tRNA biosynthesis; e, nicotinate and nicotinamide metabolism.

Based on the 39 identified metabolites, pathway enrichment analysis was conducted using the online analysis platform—MetaboAnalyst. As shown in Figure 4 and Table 2, five metabolic pathways were significantly enriched (*p*-value < 0.05). However, only the alanine, aspartate and glutamate metabolism was possibly the main disturbed metabolic pathway related to AD with an impact value >0.1 (Arima et al., 2020; Zhao et al., 2020; Sangpong et al., 2021). Notably, tryptophan metabolism had also been detected, but did not reach statistical significance (*p* = 0.56799, impact value = 0.10493).

**TABLE 2:** Significantly enriched pathways related to alcohol dependence (AD) based on 39 screened metabolites.

Pathway name	Hits/Total	The detected metabolite (KEGG identifier)	*p*-value	–log_10_(*p*) value	FDR	Impact
Alanine, aspartate and glutamate metabolism	3/28	l-Aspartic acid (C00049)	0.016922	1.7715	0.35537	0.27164
		Asparagine (C00152)				
		Oxoglutarate (C00026)				
Histidine metabolism	3/16	l-Aspartic acid (C00049)	0.0034037	2.4681	0.14295	0.04918
		Formiminoglutamic acid (C00439)				
		Imidazoleacetic acid (C02835)				
Arginine biosynthesis	3/14	l-Aspartic acid (C00049); Oxoglutarate (C00026)	0.0022734	2.6433	0.14295	0
		N-Acetylornithine (C00437)				
Aminoacyl-tRNA biosynthesis	4/48	Asparagine (C00152)	0.013778	1.8608	0.35537	0
		Glycine (C00037); l-Aspartic acid (C00049)				
		Leucine (C00123)				
Nicotinate and nicotinamide metabolism	2/15	l-Aspartic acid (C00049); Quinolinic acid (C03722)	0.034579	1.4612	0.58093	0

Hits, the number of the differential metabolites detected in a given metabolic pathway; Total, the number of all metabolites in a given metabolic pathway; KEGG, Kyoto Encyclopedia of Genes and Genomes; FDR, false discovery rate.

### Classifier Model Construction and Evaluation for Discrimination of Alcohol-Dependent Inpatients

**FIGURE 5:**
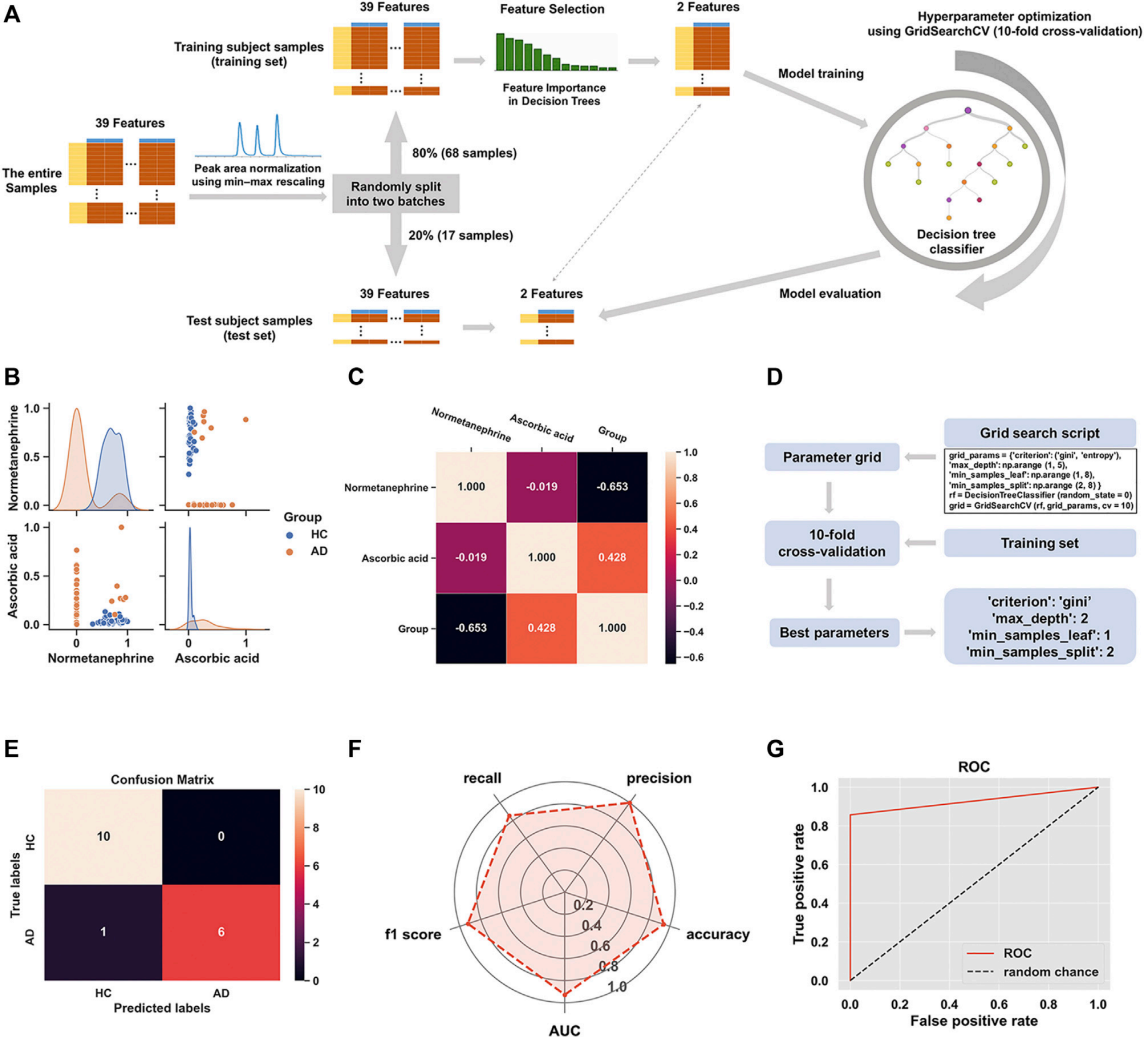
**(A)** The flowchart of the construction of decision tree classifier model; **(B)** The scatter plot of normetanephrine versus ascorbic acid, along with a layered kernel density estimate for the marginal plots along the diagonal; **(C)** A heatmap visualization of the overall correlations between the groups and the two features based on the Spearman’s correlation coefficient; **(D)** Implementation of grid search with ten-fold cross-validation on the training set; **(E)** The confusion matrix visualization (a 2 × 2 table) summarizing the prediction results of our classification model. The lower right, upper left, upper right, and lower left tables refer to the True Positive (TP), True Negative (TN), False Positive (FP), and False Negative (FN), respectively. The other metrics derived from the confusion matrix are as follows: accuracy (calculated as [TP + TN]/[TP + TN + FP + FN]), precision (calculated as TP/[TP + FP]), sensitivity/recall (calculated as TP/[TP + FN]), and f1 score (calculated as 2 × precision × recall/[precision + recall]). **(F)** The radar chart displaying evaluation metrics, including accuracy, precision, recall, f1 score, and the area under the curve (AUC); **(G)** The receiver operating characteristic (ROC) curve of the decision tree classifier model for the test set with the AUC value of 0.929.

**FIGURE 6:**
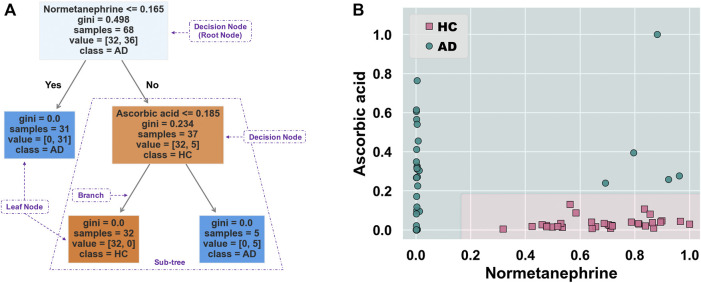
**(A)** Decision tree structure fitted on the training set. This tree-structured classifier consists of decision nodes (representing the features), branches (representing the decision rules), and leaf nodes (representing the outcome). The “Gini index” is used as an attribute selection measure for the nodes to create split points, thus implementing a decision tree. It is calculated as $\left(1-\sum_{i=1}^{m}P_{i}^{2}\right)$ , where *P*_*i*_ denotes the probability that a tuple belongs to class C_*i*_; the value of zero indicates that the predictive results can be determined. The “samples” represent the number of samples contained in a parent node, whereas the “value” represents the number of samples of its left and right child nodes. **(B)** The decision boundary along with the colored data points that describe the respective class labels. The line of demarcation, also called a decision surface, helps understand how the decisions are made by a decision tree classifier.

Figure 5A shows the schematic workflow for the decision tree classifier model construction and evaluation. The relative feature importance of the 39 differential metabolites was ranked as follows: normetanephrine (1.0000), ascorbic acid (0.3427), and the remaining metabolites (0.0000). Thus, two significantly distinctive metabolites (i.e., normetanephrine and ascorbic acid), were added as features in the model. The normalized peak areas of these metabolites appeared to be approximately a normal distribution of values ranging from 0 to 1 (Figure 5B). The maximum peak areas of normetanephrine and ascorbic acid were 8.594080 × 10^6^ and 6.695045 × 10^6^, respectively, and the minimum peak areas were 7.652522 × 10^3^ and 1.307590 × 10^6^, respectively. Alternatively, as depicted in Figure 5C, a correlation coefficient of −0.019 indicated no obvious multicollinearity between normetanephrine and ascorbic acid. Figure 5D presents the process and script of ten-fold cross-validated grid search, yielding the main optimal parameters, as follows: 1) “criterion”: “gini”; 2) “max_depth”: 2; 3) “min_samples_leaf”: 1; and 4) “min_samples_split”: 2. The discriminant performance of our model in the test set was evaluated by a critical metric, called the confusion matrix (Figure 5E), deriving from where the following classification evaluation metrics were as follows: accuracy (0.941), precision (1.000), sensitivity/recall (0.857), and f1 score (0.923) (Figure 5F). Another evaluation metric—the ROC curve—is presented in Figure 5G and yielded an AUC value of 0.929. The high f1 score and AUC values in the test set suggested that the developed model obtained a good classifier performance in terms of relying only on two metabolites that can also be called biological correlates. The decision tree structure fitted on the training set is visualized in Figure 6A. Using the Gini impurity of the features as the splitting criteria, normetanephrine was taken as the root node, from where the tree of depth two started. A decision boundary of the fitted decision tree model was also visualized to identify the decision region signifying the two classes in the two-dimensional feature space (Figure 6B).

## Discussion

This is the first study to explore the plasma metabolic profiling and potential biological correlates of AD through the approach of combining metabolomics and interpretable machine learning. Herein, we have applied a high-throughput LC-MS/MS-based metabolomics method to discover 39 differential metabolites between AD and HC individuals and a significantly altered metabolic pathway most closely related to AD (i.e., alanine, aspartate and glutamate metabolism). In addition, normetanephrine and ascorbic acid were demonstrated as suitable biological correlates of AD patients based on an interpretable decision tree classifier model.

Ascorbic acid (i.e., vitamin C) was among the differential metabolites related to AD, identified with the highest VIP value in our study. Generally, vitamin C deficiency is common in patients with unhealthy alcohol consumption such as AUD (Lim et al., 2018; Marik and Liggett, 2019), possibly in the light of the intestinal malabsorption and insufficient hepatic transformation of vitamins caused by ethanol-induced enterocyte toxicity and hepatotoxicity (Majumdar et al., 1981; Lim et al., 2018). Interestingly, we obtained the opposite result; that is, ascorbic acid was upregulated in AD patients. A possible explanation is that some AD patients may have received dietary or short-term intravenous supplementation with vitamin C. Vitamin C can afford protection against toxic accumulation of acetaldehyde, thereby reducing endothelial dysfunction, hepatotoxicity, and the possible biochemical basis for addiction (Hipólito et al., 2015; Lim et al., 2018). 4-Pyridoxic acid, the catabolic product of vitamin B_6_, was another differential metabolite related to AD, identified with the highest FC value in this study. A previous study has demonstrated the significant correlation between inadequate vitamin B_6_ intake and the 24-h 4-pyridoxic acid excretions of 0.15 mg or less (Lewis and Nunn, 1977). Additionally, acetaldehyde can act as a responsible agent accelerating the pyridoxal 5′-phosphate (a metabolically active form of vitamin B_6_) degradation into 4-pyridoxic acid (Vech et al., 1975). This partly explained the upregulated level of 4-pyridoxic acid in AD in our study, thus indicating a possible vitamin B_6_ deficiency status, which may be a key reason for AD (Hoyumpa, 1986). Moreover, considerable evidence implicates alcohol-induced gut microbiome dysbiosis and mucosal immune system disturbances (Bode and Bode, 2003; Qamar et al., 2019). Gut microbiota also participates in synthesizing constituents of vitamin B (e.g., vitamin B_6_ and B_12_), which are essential to many enzymatic reactions such as those in the tryptophan/kynurenine pathway (Ramakrishna, 2013; Więdłocha et al., 2021). These findings indicated that gut microbiota affected by alcohol might influence vitamin B levels, thus affecting tryptophan metabolism regulation.

**FIGURE 7:**
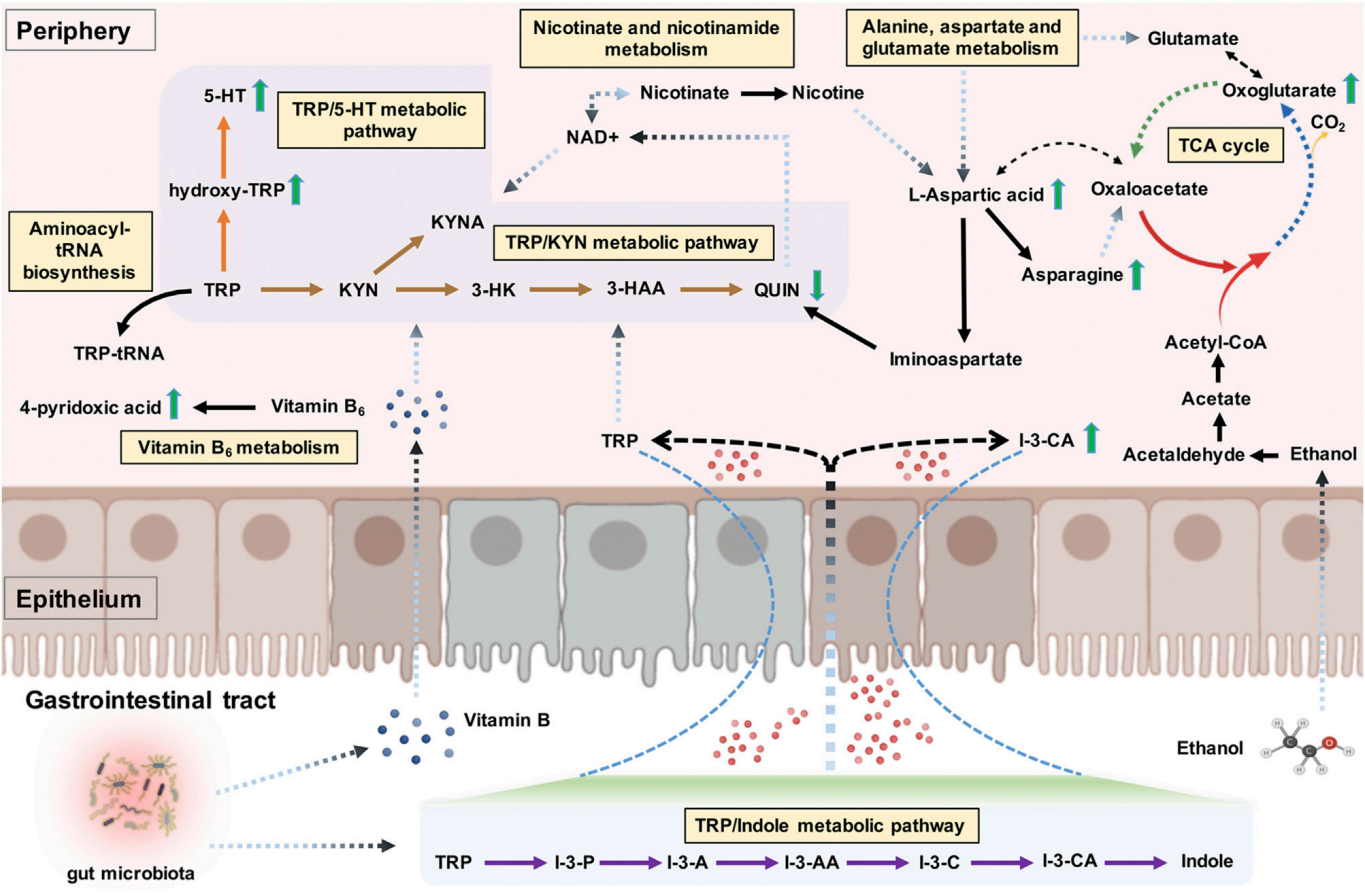
The tryptophan metabolism regulation associated with alcohol dependence (AD). The detected significant metabolite changes were represented by green up/down arrows. The tryptophan/indole, tryptophan/kynurenine, and tryptophan/serotonin metabolic pathways were donated by purple, brown, and orange arrows. The interactive influences between tryptophan metabolism and other metabolic pathways were also visualized. Note. TRP, tryptophan; I-3-P, indole-3-pyruvate; I-3-A, indole-3-acetaldehyde; I-3-AA, indole-3-acetic acid; I-3-C, indole-3-carboxaldehyde; I-3-CA, indole-3-carboxylic acid; 5-HT, serotonin; KYN, kynurenine; KYNA, kynurenic acid; 3-HK, 3-hydroxykynurenine; 3-HAA, 3-hydroxyanthranilic acid; QUIN, quinolinic acid; TCA cycle, citric acid cycle.

As a sole precursor of serotonin, tryptophan—an essential amino acid—participates the serotonin biosynthesis, which plays a crucial role in modulating the central neurotransmission. Tryptophan metabolism involves the indole pathway in bacteria and the serotonin and kynurenine pathways in humans and other mammals (Modoux et al., 2021). The kynurenine pathway accounts for above 95% of the host tryptophan metabolism, mediated by the indolamine 2,3-dioxygenase expressed in most tissues and the tryptophan 2,3-dioxygenase that is found mainly in the liver (Yamazaki et al., 1985). This leads to producing an array of downstream metabolites called “kynurenines,” including kynurenic acid, 3-hydroxykynurenine, 3-hydroxyanthranilic acid, and quinolinic acid (Zhu et al., 2021a). Studies have been conducted on the links between alcohol exposure and tryptophan metabolism, though they mainly focused on the tryptophan/serotonin pathway (LeMarquand et al., 1994; Morales-Puerto et al., 2021). Our study revealed the up-regulation of hydroxy-tryptophan and serotonin. In contrast, a down-regulation of quinolinic acid in AD patients indicates that the host tryptophan metabolism was probably more inclined to the tryptophan/serotonin pathway in AD patients than in healthy individuals. Indole-3-carboxylic acid, an indolic compound derived from the bacterial metabolites of tryptophan (Konopelski et al., 2019), was also found to be elevated in the plasma samples of AD patients. A branch of the tryptophan metabolic fate through the bacterial pathway is to be transaminated to indole-3-pyruvate, transformed to series downstream indole derivatives such as indole-3-acetaldehyde, indole-3-acetic acid, indole-3-carboxaldehyde, and indole-3-carboxylic acid, followed by spontaneous decarboxylation of indole-3-carboxylic acid to indole (Lübbe et al., 1983; Agus et al., 2018). Our findings suggested a potential regulatory role of gut microbiota in dietary tryptophan metabolism in AD, possibly referring to the changes in gut permeability (Leclercq et al., 2014; Zhu et al., 2021b). A visual summary of the changes of these detected significantly differential metabolites related to tryptophan metabolism is shown in Figure 7.

Despite having other undetected kynurenine pathway metabolites, such as kynurenic acid, the neuromodulatory roles of the kynurenine pathway metabolites (particularly the kynurenic acid) in the brain circuits related to addiction have been receiving more attention recently (Morales-Puerto et al., 2021). For example, kynurenic acid could counteract the drug abuse-associated addictive effects by regulating glutamatergic transmission *via* acting at several potential receptors on the brain, such as the N-Methyl-d-Aspartate (NMDA) receptor (Morales-Puerto et al., 2021). Moreover, given that the imbalance of neuroprotective and neurotoxic kynurenine pathway metabolites is associated with the pathogenesis of neuropsychiatric disorders (Myint et al., 2007; Muneer, 2020; Zhu et al., 2021a), the disturbances of tryptophan metabolism along the kynurenine pathway may contribute to the co-occurrence of alcohol exposure and mental disorders in the context of addiction (Neupane et al., 2015; Jiang et al., 2020; Vidal et al., 2020).

To our surprise, tryptophan metabolism was not significantly enriched; conversely, alanine, aspartate, and glutamate metabolism was identified as the main abnormal, enriched metabolic pathway related to AD. These results were partly in accordance with a previous metabolic study that reported significantly altered metabolic pathways in AUD subjects, including aspartate/asparagine metabolism, glutamate metabolism, tryptophan metabolism, and histidine metabolism (Obianyo et al., 2015). Alcohol consumption is commonly associated with the metabolite profile changes in lipids and weak organic acids, many of which are important for energy metabolism (Voutilainen and Kärkkäinen, 2019). The citric acid cycle (TCA cycle) allows the release of stored energy through the oxidation of acetyl-CoA to CO_2_, a precursor for several amino acids (e.g., alanine, glutamate, aspartate, and asparagine) (Figure 7). An imbalance in energy metabolism may result in the generation of intracellular reactive oxygen species and the accumulation of toxic metabolites and ultimately lead to metabolic diseases. The polymorphisms of alcohol dehydrogenase (ADH) and aldehyde dehydrogenase (ALDH2) are the most well-established genetic factors related to AD (Wang et al., 2012). For example, the ALDH2*2 allele, found almost exclusively among Asians, has been shown to reduce the risk for AD (Wall, 2005). ADH is mostly located in the cytosol of the hepatocyte and involves metabolizing alcohol to acetaldehyde, which is further metabolized by ALDH2 to produce acetate in the mitochondria (Cederbaum, 2012). Alcohol metabolism exerts epigenetic effects via several mechanisms, including the formation of acetate. In cells with mitochondria such as the brain, the acetate can be transformed by enzymes to acetyl-CoA, which is used in histone acetylation, thus resulting in gene activation (Zakhari, 2013). The acetate is eventually metabolized to CO_2_ *via* the TCA cycle, thus generating energy and providing precursors essential for amino acid biosynthesis (Figure 7). Mounting evidence suggests that heavy alcohol exposures decrease brain glucose metabolism but facilitate the use of acetate as an alternative brain energy source in the human brain (Volkow et al., 2013), indicating that a ketogenic diet may be an effective treatment for easing alcohol withdrawal symptoms in humans (Dencker et al., 2018).

In this study, we particularly focused on the distinctive metabolites and significantly enriched metabolic pathways related to tryptophan metabolism regulation. l-aspartic acid was the most involved regarding the nine detected distinctive metabolites included in the significantly enriched metabolic pathways. As one example, l-aspartic acid is a non-essential amino acid, which plays an important role in synthesizing other amino acids such as asparagine, methionine, arginine, isoleucine, and lysine, and also serves as a neurotransmitter acting at the glutamate receptor (Downing et al., 1996). Besides, Hinton et al. Hinton et al. (2017) found l-aspartic acid as a metabolomics biomarker for predicting acamprosate treatment response in AD patients, suggesting l-aspartic acid as a potential biomarker for pharmaceutical response and disease discrimination in AD. Gamma-amino butyric acid (GABA) and NMDA receptors are two major receptors involved in AD, which are also believed to be important targets of alcohol (Peoples and Weight, 1999; Banerjee, 2014). Besides l-aspartic acid, glycine, glutamate, and d-serine can act as cofactors regulating the activity of the NMDA receptor (Zorumski and Izumi, 2012). The exact contributions of these amino acid cofactors to the activity of the NMDA receptor modulated by alcohol remain unclear (Ron and Wang, 2009). Nonetheless, we can speculate that this might be associated with the NMDA receptor regulation of these cofactors, and l-aspartic acid might also take part in the NMDA receptor regulation of neuroprotective and neurotoxic kynurenine pathway metabolites. Specifically, nicotinate and nicotinamide metabolism was another significantly enriched pathway involving the detected distinctive metabolites of l-aspartic acid and quinolinic acid, both implicated in the nicotinamide adenine dinucleotide (NAD+) (a metabolically active form of vitamin B_3_) biosynthetic pathway. NAD+ can reduce the acetaldehyde production and the formation of reactive oxygen species, thereby ameliorating alcohol-related organ damage (Zakhari, 2013; Xu et al., 2019b). It also serves as an essential cofactor for hundreds of enzymes (e.g., dehydrogenases) and a coenzyme in various energy metabolism pathways linked with the immune regulation of kynurenines (Savitz, 2020; Covarrubias et al., 2021); in turn, the sole *de novo* pathway for NAD+ biosynthesis is the kynurenine pathway, as quinolinic acid is the endogenous source of NAD+ (Castro-Portuguez and Sutphin, 2020). As another significantly enriched pathway, aminoacyl-tRNA biosynthesis also involves in the tryptophanyl-tRNA biogenesis via tryptophanyl-tRNA synthetase; tryptophan depletion, on the other hand, modulates the extracellular tryptophanyl-tRNA synthetase-mediated high-affinity tryptophan uptake into cells (Yokosawa et al., 2020). The interactive influences between tryptophan metabolism and different significantly enriched metabolic pathways are shown in Figure 7.

Normetanephrine was also defined as the root node, the most important splitting feature, based on the generated decision tree structure. Previous studies have demonstrated that normetanephrine, a critical neurotransmitter mediator of drug reward and the addiction process, plays a potential role in ethanol-induced self-administration and locomotion (Weinshenker and Schroeder, 2007). Patker et al. (2004) found that alcohol-dependent individuals who were actively drinking showed significantly higher normetanephrine concentrations than those in remission and healthy controls. However, alterations in normetanephrine activity appear to normalize by late alcohol withdrawal (Patkar et al., 2003). Similarly, our study showed downregulated levels of normetanephrine in AD patients compared to those in controls. A possible explanation for this finding was that the AD patients had a longer period of abstinence compared with controls. Understanding the mathematics behind the generated decision tree is straightforward. The decision nodes are tests on a feature. For example, normetanephrine has a control statement (normalized peak area of 0.165 or less); the samples satisfying this condition are on one side, while the remaining samples are on the other. They continue splitting until the leaf nodes represent the classes. Therefore, the decision tree visualization is simple to illustrate how classification is predicted by the underlying data, thus highlighting our key insights.

Our study should be considered in light of several limitations. First, our OPLS-DA model indicated a certain variation in the measured data within the AD group. A reasonable explanation invokes the difference of other factors in this group, including the period of alcohol abstinence, the frequency of smoking, and concomitant medications. For example, although the patients had not drunk alcohol since they were hospitalized, the alterations in the kinetics of the metabolites influenced by recent alcohol use might have affected the detected levels (Voutilainen and Kärkkäinen, 2019). As presented in Figure 7, ethanol intake may influence the metabolism of many amino acids (e.g., l-aspartic acid and glutamate) and the metabolic pathway—alanine, aspartate, and glutamate metabolism. Smoking, which is common among drinkers, is another confounding factor. Nicotine exposure can induce metabonomic alterations (e.g., increase of the brain levels of both excitatory and inhibitory amino acids, including aspartate, glutamate, arginine, taurine, and alanine) (Kashkin and De Witte, 2005). Concomitant medications, such as fat- and water-soluble vitamins, may also confound our findings. Future studies may focus on the subgroup analysis of AD to minimize the confounding effects of these factors. Second, although absolute quantification was not involved in our study and the widely targeted metabolomics can act as an alternative method to achieve accurate quantification of metabolite levels using semi-quantitative analysis, the optimal MS parameters may need to be validated by using the available chemical standards. Despite the possible changes of metabolite concentrations in different analysis batches or institutions, peak area normalization may minimize the influence on the classifier model. Finally, more samples may be needed for further metabolomics analysis and the development and evaluation of our machine learning model. Moreover, the samples collected from plasma may not directly reflect the brain metabolite levels, thus further research is needed to establish the relationship between blood and brain metabolites (Hinton et al., 2017).

## Conclusion

This study comprehensively analyzed plasma metabolic profiling and potential biological correlates *via* the integration of metabolomics and interpretable machine learning. Our findings suggested that vitamin deficiency status may be common in AD, particularly the vitamins B, affecting tryptophan metabolism regulation. Indole-3-carboxylic acid, quinolinic acid, hydroxy-tryptophan, and serotonin were identified as significantly distinctive metabolites related to AD, involving the tryptophan metabolism along the indole, kynurenine, and serotonin pathways. Alanine, aspartate and glutamate metabolism was identified as the main abnormal, enriched metabolic pathways associated with AD. We found that tryptophan metabolism interactively influenced other metabolic pathways, such as nicotinate and nicotinamide metabolism. Using a decision tree classifier model, normetanephrine and ascorbic acid were demonstrated as suitable biological correlates of AD. Nevertheless, normetanephrine was identified as the most important feature. l-aspartic acid involved multiple significantly enriched pathways and the possible NMDA receptor regulation of kynurenines. Future studies should focus on the global analysis of the possible roles of these differential metabolites and disordered metabolic pathways in the pathophysiology of AD.

## Data Availability

The raw data supporting the conclusion of this article will be made available by the authors, without undue reservation.
