# Supplementary material for: Combining Metabolomics and Interpretable Machine Learning to Reveal Plasma Metabolic Profiling and Biological Correlates of Alcohol-Dependent Inpatients: What About Tryptophan Metabolism Regulation?
Source: Front Mol Biosci. 2021 Nov 8;8:760669. doi: 10.3389/fmolb.2021.760669 (PMC8630631; doi:10.3389/fmolb.2021.760669)
Supplement: Supplementary file 1 [file DataSheet1.PDF]

Table S1 The names of 59 and 52 compounds that semi-quantitatively measured in positive and negative ionization modes.

Positive ionization mode	Negative ionization mode
1-Methyl-Histidine	1,3-Diphopshateglycerate
3-OH-Anthranilate	2-Deoxyglucose-6-Phosphate
5-Hydroxy Indole Acetic Acid	2-Hydroxy-2-Methylbutanedioic Acid
Acetylcarnitine	2-Hydroxygluterate
Acetyllysine	2-Isopropylmalic Acid
Adenine	2-Ketohaxanoic Acid
Arginine	4-Pyridoxic Acid
Asparagine	5-Phosphoribosyl-1-Pyrophosphate
Aspartate	Aconitate
Carnitine	ADP
Citrulline	Allantoate
Creatine	Allantoin
Creatinine	Ascorbic Acid
Cysteamine	Carbamaoyl Asparatate
Dimethylglycine	Carbamoyl Phosphate
Dopamine	Cholesteryl Sulfate
Formiminoglutamic Acid	Citraconic Acid
Glucosamine	Citrate
Glutamate	dGDP
Glycerophosphocholine	D-Gluconate
Glycine	d-Glucose
Hydroxyproline	dGTP
Hydroxy-Tryptophan	Dihydroorotate
Imidazoleacetic Acid	Fructose-1,6-Bisphosphate
Inosine	Glucono-D-Lactone
Inosione	Glycerate
L-Alanine	Hexose Phosphate
L-Alpha-Aminobutyrate	Hydroxyisocaproic Acid
L-Arginine	Hydroxyphenylpyruvate
L-Aspartic Acid	Indole-3-Carboxylic Acid
Leucine	Indoleacrylic Acid
L-Glutamine	Isocitrate
L-Histidine	Lactate
L-Ornithine	Malate
L-Proline	Methylmalonic Acid
L-Serine	Myo-Inositol
L-Threonine	N-Acetyl-L-Alanine
L-Tyrosine	Oxoglutarate

Mesaconic Acid	Pantothenate
Metanephrine	Pga
Methionine	Phenyllactic Acid
Methionine Sulfoxide	Phenylpropiolic Acid
Methylcysteine	Phosphoribosyl Pyrophosphate
Methylnicotinamide	Pyroglutamic Acid
Methylsuccinic Acid	Pyrophosphate
N-Acetyl-Glutamine	Ribose-Phosphate
N-Acetylornithine	Sedoheptulose 1,7-Bisphosphate
N-Carbamoyl-Beta-Alanine	Succinate
Normetanephrine	Taurine
O-Acetyl-L-Serine	UdP
Phenylacetylglutamine	Uric Acid
Pipecolic Acid	Uridine
Quinolinic Acid	
Sarcosine	
Serotonin	
Spermine	
Tryptamine	
Urea	
Valine	

Table S2 The differential metabolites with variable influence on projection (VIP) values >1 and *P*-value < 0.05 of t-test after false discovery rate (FDR) adjusting in the OPLS-DA model.

Compound name	VIP	*P*-value	FDR
Ascorbic Acid	2.350	3.93E-29	4.36E-27
Hexose Phosphate	2.024	2.09E-12	6.80E-11
Phenylpropiolic Acid	1.964	1.44E-13	8.01E-12
Pantothenate	1.923	2.45E-12	6.80E-11
Dihydroorotate	1.834	1.76E-11	3.91E-10
Normetanephrine	1.809	5.12E-10	7.10E-09
Indole-3-Carboxylic Acid	1.752	3.22E-10	5.11E-09
4-Pyridoxic Acid	1.744	2.37E-08	2.63E-07
Glucosamine	1.692	2.80E-10	5.11E-09
Taurine	1.638	4.13E-08	4.17E-07
L-Aspartic Acid	1.582	1.08E-08	1.33E-07
O-Acetyl-L-Serine	1.531	2.67E-07	2.28E-06
Glucono-D-Lactone	1.438	5.35E-07	4.24E-06
Oxoglutarate	1.422	5.04E-08	4.66E-07
Allantoin	1.413	3.35E-06	2.33E-05

Imidazoleacetic Acid	1.387	2.04E-06	1.51E-05
Asparagine	1.364	5.40E-06	3.53E-05
Quinolinic Acid	1.320	7.31E-06	4.25E-05
Leucine	1.316	8.96E-06	4.73E-05
Glycine	1.299	5.95E-06	3.67E-05
Metanephrine	1.284	1.13E-05	5.70E-05
Malate	1.275	7.65E-06	4.25E-05
Dimethylglycine	1.274	1.76E-05	8.13E-05
Pipecolic Acid	1.263	2.07E-05	9.19E-05
Formiminoglutamic Acid	1.224	3.82E-05	0.00016289
2-Deoxyglucose-6-Phosphate	1.199	0.00014154	0.00054595
d-Glucose	1.186	0.00024322	0.00087088
Sedoheptulose 1,7-Bisphosphate	1.174	1.71E-05	8.13E-05
1,3-Diphopshateglycerate	1.171	0.00014264	0.00054595
UdP	1.154	0.00010522	0.00043258
Fructose-1,6-Bisphosphate	1.136	0.00078412	0.0023554
2-Isopropylmalic Acid	1.096	0.00057782	0.0018864
L-Alpha-Aminobutyrate	1.094	0.00026008	0.00090214
Acetyllysine	1.087	0.00059833	0.0018976
2-Ketohaxanoic Acid	1.075	0.00078514	0.0023554
N-Acetylornithine	1.062	0.00037464	0.0012601
Serotonin	1.054	0.00094842	0.0026319
Hydroxy-Tryptophan	1.027	0.0001491	0.00055169
Methylcysteine	1.008	0.0020791	0.0049103
